# Natural Health Products for Anti-Cancer Treatment: Evidence and Controversy

**DOI:** 10.3390/jpm14070685

**Published:** 2024-06-26

**Authors:** Valeria Conti, Giovanna Polcaro, Emanuela De Bellis, Danilo Donnarumma, Federica De Rosa, Berenice Stefanelli, Graziamaria Corbi, Francesco Sabbatino, Amelia Filippelli

**Affiliations:** 1 Department of Medicine, Surgery, and Dentistry, Scuola Medica Salernitana, University of Salerno, 84081 Baronissi, Italy; vconti@unisa.it (V.C.); gpolcaro@unisa.it (G.P.); fderosa@unisa.it (F.D.R.); bstefanelli@unisa.it (B.S.); fsabbatino@unisa.it (F.S.); afilippelli@unisa.it (A.F.); 2 Clinical Pharmacology Unit, San Giovanni di Dio e Ruggi d’Aragona University Hospital, 84131 Salerno, Italy; 3 PhD School “Clinical and Translational Oncology (CTO)”, Scuola Superiore Meridionale, University of Naples “Federico II”, 80138 Naples, Italy; d.donnarumma@ssmeridionale.it; 4 Department of Translational Medical Sciences, University of Naples “Federico II”, 80131 Naples, Italy; graziamaria.corbi@unina.it; 5 Oncology Unit, University Hospital “San Giovanni di Dio e Ruggi d’Aragona”, 84131 Salerno, Italy

**Keywords:** antioxidants, nutraceutical, natural products, vitamins, cancer

## Abstract

Natural Health Products (NHPs) have long been considered a valuable therapeutic approach for the prevention and treatment of various diseases, including cancer. However, research on this topic has led to inconclusive and often controversial results. This review aims to provide a comprehensive update of the effects and mechanisms related to the use of NHPs, to describe the results of randomized clinical trials (RCTs) on their effects in cancer patients, and to critically discuss factors influencing clinical outcomes. RCTs available in the literature, even those studying the same NHP, are very heterogeneous in terms of indications, doses, route and timing of administration, and outcomes evaluated. Silymarin, ginsenoside, and vitamin E appear to be useful in attenuating adverse events related to radiotherapy or chemotherapy, and curcumin and lycopene might provide some benefit in patients with prostate cancer. Most RCTs have not clarified whether NHP supplementation provides any real benefit, while harmful effects have been shown in some cases. Overall, the available data suggest that although there is some evidence to support the benefits of NHPs in the management of cancer patients, further clinical trials with the same design are needed before their introduction into clinical practice can be considered.

## 1. Introduction

Natural Health Products (NHPs), such as dietary supplements, probiotics and vitamins, belong to the group of unconventional practices called Complementary and Alternative Medicine (CAM).

CAM is considered a valuable approach in several clinical settings because of its alleged ability to positively influence the response to drug therapy in terms of efficacy and safety and to improve patients’ quality of life (QOL) [1,2,3].

**Figure 1:**
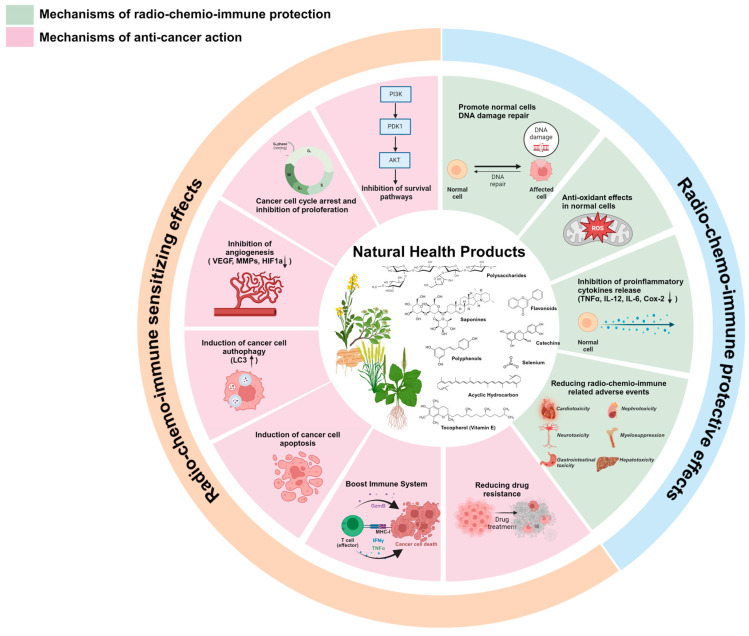
Mechanisms of anti-cancer action and radio–chemo immune protection of chemical classes isolated from Natural Health Products to improve the efficacy of cancer therapy and mitigate related side events.

In the continuously evolving landscape of cancer treatment, NHPs have garnered considerable attention as potential therapeutic agents for cancer treatment, either as single agents or in combination with conventional therapies. NHPs are usually obtained from plants, marine organisms and microorganisms, and display a wide range of chemical structures with different pharmacological activities [4]. These include the inhibition and modulation of tumorigenic pathways involved in cancer pathogenesis such as cell proliferation, angiogenesis, metastasis and apoptosis evasion. Moreover, most of these compounds have a relatively low toxicity profile. As a result, their characteristics makes them attractive candidates for therapeutic development [5]. Polyphenols, alkaloids, flavonoids and terpenoids are the most extensively studied NHPs that, due to their diverse chemical structures and biological activities, have shown promising anti-cancer properties in preclinical studies and early-phase clinical trials [6]. Several randomized clinical trials (RCTs) have been conducted using NHPs to improve the efficacy of radio–chemo–immuno-based therapy and to mitigate related side effects or protect normal cells from iatrogenic toxicity (Figure 1). However, mixed and inconclusive results have been reported [7,8,9,10]. 

Variations in study design, patient characteristics, dosage and formulation of natural products, concomitant treatments and outcome measures have negatively influenced the results of the RCTs. Consequently, a thorough analysis of factors influencing clinical outcomes is essential to accurately interpret study findings and to pave the way for personalized treatment approaches that incorporate NHPs into cancer therapy. 

In this review we aim to provide a comprehensive update of the effects and mechanisms related to the use of NHPs in cancer, to describe the results of randomized clinical trials (RCTs) available in the literature in the last two decades, and to provide a critical analysis of the factors influencing clinical outcomes critically evaluating the impact of these factors on the interpretation of RCTs.

## 2. Investigating the Efficacy and Mechanisms of NHPs in Enhancing Cancer Radiotherapy

Radiotherapy is a cornerstone of cancer treatment that uses ionizing radiation to induce DNA damage, triggering tumor cell death. While effective, radiotherapy can inadvertently damage surrounding healthy tissues, leading to differential adverse events [11,12]. Recent preclinical and clinical studies have clearly demonstrated that NHP supplementation to radiotherapy can potentially increase treatment efficacy (radiosensitizing effects) or protect normal cells from radiation-induced damage (radioprotective effects), improving patients’ clinical outcomes [13]. In particular, some NHPs have been shown to exhibit potent radiosensitizing effects acting on key molecular pathways involved in tumor radioresistance and regulation of cell proliferation, DNA damage, survival, induction of apoptosis, and cellular responses to stress and inflammation including PI3K/Akt/mTOR, miR-34a/Sirt1/p53, MAPK and NF-kB pathways [14,15,16]. Among the various natural compounds, curcumin (a polyphenolic compound derived from turmeric Curcuma longa), resveratrol (a stilbenoid compound derived from grapes, berries or peanuts), epigallocatechin-3-gallate (EGCG) (the predominant catechin in green tea) and quercetin (a potent antioxidant flavonoid plant pigment) have been shown to inhibit PI3K/Akt/mTOR and NF-kB pathways, significantly increasing radiotherapy-induced cancer cell death [13,16,17,18,19,20,21,22]. In addition, besides inducing apoptosis through the PI3K/Akt/mTOR and NF-kB pathways, EGCG and curcumin could also exert radiotherapy-induced cancer cell death by activating the miR-34a/Sirt1/p53 signaling pathway or inhibiting the MAPK pathway, respectively [13]. All of these compounds, along with others, also showed a potent radioprotective effect by promoting DNA damage repair and anti-oxidant/anti-inflammatory activity [11,13]. Specifically, curcumin, resveratrol, EGCG, quercetin, apigenin (a natural plant flavonoid found in parsley, celery or chamomile tea) and genistein (an isoflavone derived mainly from soy) have been shown to promote DNA damage repair and integrity through activation of DNA repair enzymes and attenuation of oxidative stress, respectively [11,13]. In contrast, astragalus and schisandra (polysaccharides isolated from Astragalus membranaceus and Schisandra chinensis [23], Hoehenbuehelia serotina (a species of fungus), ginsenoside and acanthopanax senticosus (saponins derived from ginseng), matrine, ligustrazine and β-carboline (alkaloids derived from Ligusticum chuanxiong, Peganum harmala and Banisteriopsis caapi) [11,13], vitamin C (primarily found in oranges, lemons, kiwi, strawberries, peppers, broccoli and cabbage) and E (present in oily nuts, almonds, wheat germ oil and vegetable oils), selenium (an essential trace element found in foods like Brazil nuts, tuna, chicken, turkey, beans, lentils and eggs) and carotenoids (found in carrots, spinach, pumpkin, melons and tomatoes) have a recognized ability in scavenging free radicals generated by ionizing radiation [6]. Finally, curcumin, genistein, hesperidin (a flavonoid found in citrus fruits), ferulic acid (a phenolic compound found in grains, fruits and vegetables) and caffeine (found in coffee or tea) have been linked to down-regulation of inflammatory cytokines or inflammatory mediators, including TNF-α, IL-6, IL-12 and Cox-2, thereby reducing radiotherapy-induced inflammation [11,13].

## 3. Synergistic Enhancement of Cancer Chemotherapy by NHPs: Mechanisms and Clinical Implications

Chemotherapy, a widely used cancer treatment, relies on cytotoxic drugs that target rapidly growing cancer cells. However, chemotherapy often induces systemic toxicity and drug resistance, limiting its effectiveness [24]. Preclinical studies and clinical trials have shown that NHPs integrated into chemotherapy regimens can enhance the tumor-killing effect by reducing the development of drug resistance (chemosensitization effects) or mitigate chemotherapy-induced side effects (chemoprotective effects). Several natural compounds have been shown to exert potent chemosensitization effects by inhibiting inflammation, tumor proliferation and angiogenesis, as well as inducing apoptosis/necrosis and autophagy of cancer cells [25]. For example, curcumin, resveratrol, naringin (a natural bioflavonoid derived from grapefruit or other citrus fruits) and berberine, an isoquinoline alkaloid found in several medicinal plants, including Berberis vulgaris, Coptis chinensis, Hydrastis Canadensis, and Berberis aristata [26], can inhibit the expression of Cox-2 and NF-kB, both involved in inflammatory signaling pathways [27,28]. Curcumin, resveratrol, naringin and berberine can also block cell division by inhibiting cyclin-dependent kinases (CDKs) and other cell-cycle regulatory proteins [27,28,29]. They can also restrain the secretion of vascular growth factors and components of the signaling pathway involved in angiogenesis [28]. Curcumin, resveratrol, naringin and berberine have been shown to inhibits growth factor receptors, thereby sensitizing cancer cells to apoptosis induction [28]. On the other hand, Solanum nigrum Linn. (a medicinal plant) and hederagenin (a triterpenoid isolated from Hedera helix) can promote the conversion of LC3 (microtubule-associated proteins 1A/1B light chain 3B) from its cytosolic form (LC3-I) to its lipidated form (LC3-II), thereby facilitating the formation of autophagosomes [30,31]. 

It has also been shown that some NHPs can overcome chemotherapy resistance by inhibiting components of the pro-tumorigenic pathway, drug efflux pumps, and the modulation of drug metabolism. Specifically, solamargine (a bioactive alkaloid derived from Solanum nigrum) and sulforaphane (an isothiocyanate derived from cruciferous vegetables) can modulate the Hedgehog pathway [32]; tagitinin C (a sesquiterpenoid compound isolated from the Tithonia diversifolia) and ginkgetin (a biflavonoid isolated from Ginkgo biloba) target the Nrf2/HO-1 pathway [33]. EGCG inhibits drug efflux pumps and the modulates drug metabolism pathway [28,29,30,31,32,33,34]. 

The chemoprotective effects of NHPs are related to their free-radical scavenger properties, ability to inhibit components of the inflammatory pathway or apoptosis induction, activation of cholinergic neurotransmission and stimulation of bone marrow cells, and a neuroprotective and cardioprotective role. In particular, gingerol (a phenolic phytochemical compound found in fresh ginger) and silymarin (a mixture of flavonolignans isolated from milk thistle plant Silybum marianum) may reduce chemotherapy-induced liver damage by scavenging free radicals and inhibiting inflammatory pathways [35,36]. Gingerol seems also able to combat chemotherapy-induced nausea, vomiting, myalgia and insomnia by acting on serotonin release and on cholinergic (M3) receptor activities [37,38]. Berberine, tanshinone IIA (a lipophilic active constituent isolated from Salvia), geraniol (a monoterpenoid alcohol present in essential oils) and thymoquinone (a benzoquinone isolated from Nigella sativa) could contrast chemotherapy-induced neurotoxicity and neuroinflammation by inhibiting the expression of apoptosis-related proteins (p53, MAPK, etc.) in neuronal cells and increasing brain AchE activity [32]. Thymoquinone, as reported for curcumin and resveratrol, could attenuate chemotherapy-induced nephrotoxicity by increasing the NAD+ dependent Sirt1, which in turn reduces the activation of the chemotherapy-associated p53 acetylation and apoptosis induction [32]. Ginsenoside Rg3 (a triterpenoid saponin found in ginseng) can mitigate chemotherapy-induced bone marrow suppression by promoting the proliferation of total spleen and bone-marrow cells (BMCs) [39]. Hesperidin is reported to reduce diarrhea-related chemotherapy by inhibiting the expression of inflammatory factors, as well as by suppressing STAT3 activity in intestinal tissues [32]. Finally, quercetin, silymarin, calycosin (an isoflavone found in Astragalus membranaceus), hydroxytyrosol (olive oil phenolic antioxidant) and colchicine (an alkaloid extracted from Colchicum and found in corn, seeds or flowers) can exert a cardioprotective role on chemotherapy effect by inhibiting the NLRP3-cystatin-1-GSDMD pathway and oxidative stress in cardiomyocytes [32,40].

## 4. Unveiling the Potential of Natural Compounds in Boosting Immunotherapy: Mechanisms and Clinical Implications

Immunotherapy has emerged as a revolutionary approach in cancer treatment, harnessing the body’s immune system to target and eliminate cancer cells. Despite its notable successes in specific cancer types, persistent challenges include limited response rates and the occurrence of immune-related adverse events [41,42,43]. To date, there are a limited number of preclinical studies or early-phase clinical trials evaluating the effect of NHPs in combination with immunotherapy. These studies have shown that NHPs have the potential to enhance and reduce, respectively, the efficacy and toxicity of immunotherapy through immunomodulating activity. Specifically, curcumin, ginseng, astragalus membranaceus extracts, quercetin, gambogic acid (a flavonoid compound extracted from the resin of the Garcinia hanburyi) and baicalin (a flavone glycoside extracted from Scutellaria baicalensis) have been shown to increase immune cell infiltration into tumors and boost T, B, dendritic and natural killer (NK) cell activity by promoting the production of cytokines (e.g., IL-6, IL-12, TNF-α and IFN-γ) or antibodies, as well as down-regulating immune checkpoints (e.g., CTLA-4, Foxp3 or PD-L1), thereby inhibiting tumor progression and metastasis [44,45,46]. On the other hand, these compounds can also exert an immunosuppressive effect by inhibiting regulatory T cells (Tregs) and myeloid-derived suppressor cells (MDSCs) by reducing the production of immunosuppressive cytokines (e.g., IL-10 and TGFß) or the activation of STAT3 signaling [44], respectively. Consequently, by modulating inflammatory responses, these compounds have also been associated with the attenuation of inflammatory damage to healthy tissues resulting from generalized immune activation. Similarly, plantain polysaccharide (PLP), extracted from the whole plant of Plantago asiatica L., has been shown to promote dendritic cell maturation, M1 phenotype macrophage polarization and intratumoral matrix remodeling by modulating cytokine release (e.g., IL-12 p70, TNF-α, IL-1β, and IL-6), M1 surface molecule expression (e.g., CD80 and CD86) and nitric oxide production, respectively [45].

## 5. Vitamins and Other Micronutrients in Cancer Patients

Vitamins and other micronutrients are frequently used by patients with hematologic malignancies and solid tumors [47].

Vitamin E is a fat-soluble vitamin of the Tocotyol family (including four tocopherols and four tocotrienols). These compounds have the role of protecting the integrity of cell membranes by inhibiting lipid peroxidation and acting as antioxidants. They are also involved in maintaining neurological structure and function, and they protect red blood cells from lysis caused by irradiation [48,49,50]. Vitamin C is involved in several physiological processes, mainly due to its electron-donating property. It plays an important role in protection from reactive oxygen species (ROS), prevention of endothelial dysfunction, modulation of gene transcription and DNA methylation [51]. Cancer patients often have lower plasma levels of vitamin C than healthy subjects. For this reason, several RCTs have been designed to evaluate the potential anticancer effect of vitamin C supplementation. As early as the 1970s, Cameron and Linus Pauling showed that high doses of intravenous and oral vitamin C significantly prolonged the survival of patients with terminal cancer [52]. Subsequently, two RCTs, based on oral administration, failed to reproduce similar results; rather, they showed no beneficial effects of high-dose vitamin C against advanced malignancies [53,54]. However, these two clinical studies are not at all comparable with the first one, mainly because of the route of administration. Indeed, intravenously administered vitamin C can reach higher plasma levels (about 70-fold) than that orally administered [55]. 

As with vitamin C, the effects of vitamin D have been studied on the assumption that its low circulating levels have been correlated with increased cancer risk [56]. Indeed, several studies have shown potential antitumor effects of vitamin D supplementation in colorectal, breast, pancreatic, ovarian, and prostate cancers. In addition, because vitamin D acts as a regulator of immune system processes, it has been suggested that it might sensitize cancer immunotherapy [56]. However, a recent meta-analysis including 14 RCTs with 104,727 participants using vitamin D supplementation failed to found a statistically significant effect in reducing cancer mortality [57]. 

Selenium is an essential mineral incorporated into selenoproteins, which, like vitamins, are involved in the body’s antioxidant defense mechanisms. Furthermore, in the immune system, selenium stimulates the formation of antibodies and the activity of T helper cells, cytotoxic T cells and Natural Killer (NK) cells [58]. Several studies have been conducted to evaluate possible benefits of selenium supplementation in cancer based on the assumption that observational studies have found that high circulating levels of selenium are associated with a lower risk of cancer in the general population [59]. However, RCTs have mostly failed to confirm the existence of these protective effects [60], suggesting, rather, an increased risk of developing specific neoplasms such as high-grade prostate cancer [61].

## 6. Randomized Controlled Trials of Natural Health Products in Cancer Patients

Table 1 and Table 2 report the characteristics and main results of RCTs available in the literature over the past two decades that have tested the effects of herbal supplements (Table 1) and vitamins and other micronutrients (Table 2) in different types of cancer. 

Among herbal supplements, RCTs have been carried out to study the potential anticancer effects of agents such as resveratrol, genistein, ginsenoside, silymarin and curcumin.

Genistein and ginsenoside have been reported to play a positive role in prostate and breast-cancer patients, respectively. Lazarevic et al. studied the possible role of supplementation with synthetic genistein used at a dosage of 30 mg for 3–6 weeks before prostatectomy. They reported a significant reduction (*p* = 0.051) in serum PSA levels in patients who received genistein compared with the placebo group, in which PSA increased by 4.4 percent [63]. In breast cancer patients, the RCT by Hamidian et al. evaluated the potential role of Panax ginseng, containing ginsenoside, in attenuating doxorubicin-induced cardiac toxicity. A significant protective role was found in patients supplemented with 1 g daily after four and eight cycles of chemotherapy (*p* < 0.001) [64]. In contrast, another RCT that evaluated the potential effects of Panax ginseng (400 mg twice daily for 28 days) in reducing fatigue in patients with various advanced malignancies (including breast cancer) found no differences in outcomes among patients who received such supplementation compared with placebo [65]. Finally, supplementation with 500 mg daily of Panax ginseng was protective (supplemented patients vs. placebo, *p* < 0.001) against radioiodine therapy-induced genotoxicity in patients with thyroid cancer [66].

No evidence of beneficial effects emerged from RCTs using resveratrol. In contrast, in patients with colorectal/hepatic metastases, administration of 5 g of micronized resveratrol daily has been correlated with the possible occurrence of gastrointestinal adverse events such as nausea and diarrhea and, less frequently, other adverse events including chills, lethargy, rash, skin irritation, and vascular redness [62].

Numerous examples of evidence have been accumulated from RCTs on the use of silymarin in the management of radio- and chemotherapy-related adverse events. Silymarin is a molecule extracted from *Silybum marianum*, known for its antioxidant, anti-inflammatory and hepatoprotective activities [131].

In breast cancer patients, daily oral administration of silymarin 140 mg appears to alleviate hepatotoxicity related to the doxorubicin/cyclophosphamide/paclitaxel chemotherapy regimen (*p* = 0.012) [67]. In patients with the same type of cancer, the use of 1% silymarin gel once daily appears to delay and attenuate the severity of radiotherapy-induced dermatitis (*p* < 0.05) [68].

Similar effects were reported in patients with head and neck cancer supplemented with silymarin (at a dosage of 420 mg daily). Such supplementation was able to reduce radiotherapy-related mucositis during 6 weeks of radiotherapy (*p* < 0.05) [69].

Elyasi et al. reported that a 1% gel of silymarin twice daily delayed the onset and reduced the grade of severity of capecitabine-related hand–foot syndrome in patients with colorectal and esophagogastric patients [70]. In contrast, Shahbazi F. et al. reported no efficacy for silymarin (420 mg daily, divided into three doses during chemotherapy) in the management of nephrotoxicity caused by cisplatin in patients with upper gastrointestinal (75%) and ovarian (21%) cancer and patients with mesothelioma (4%) [71].

Curcumin has also been studied to evaluate its protective role against radiotherapy toxicity. The RCT by Talakesh et al. found no significant effects in attenuating the severity of radiation-induced skin reactions in breast cancer patients who received 80 mg daily of curcumin compared with the placebo group during the first-to-sixth weeks of treatment. However, the difference between the intervention and placebo groups became significant after seven weeks of treatment (*p* = 0.01) [74]. In breast cancer, even a dosage of 6 g per day, tested by Ryan et al. with regard to the effect of reducing radiation-induced dermatitis, was not associated with any benefit [77].

In studies conducted in prostate cancer patients who received 3 g daily of curcumin, an increase in total antioxidant capacity (TAC), without altering the therapeutic efficacy of radiotherapy, was demonstrated compared with the placebo group (*p* < 0.05) [78]. Furthermore, in patients with prostate cancer undergoing intermittent androgen deprivation (IAD), it has been shown that oral supplementation of curcumin was associated with stopping the increase in PSA values (*p* = 0.02) [75]. In head and neck cancer, the use of 0.1% curcumin for buccal washes has been shown to be insufficient to prevent, but helpful in delaying, the onset of radiation-induced oral mucositis [73].

Lycopene is a natural antioxidant belonging to the carotenoid family and found in red and yellow fruits or plants, especially tomatoes [132]. A number of studies have tested the potential benefits of lycopene-containing dietary supplements in patients with prostate cancer [132,133].

Kucuk et al. evaluated the effects of a tomato oleoresin extract containing 30 mg of lycopene for 3 weeks in 26 male patients with clinical stage Tl or T2 prostate cancer before radical prostatectomy. After surgery, subjects in the intervention group had smaller tumors, less involvement of surgical margins and/or extra-prostatic tissues with cancer, and less widespread involvement of the prostate by high-grade prostatic intraepithelial neoplasia than the control group. PSA was lower in the intervention group than in the controls [79].

In contrast, Clark et al. failed to demonstrate clinical efficacy for daily lycopene supplementation with 15, 30, 45, 60, 90, and 120 mg/day for 1 year. No serum PSA response was observed but, rather, 37% of patients had increased levels of PSA [82]. Mariani et al. also found no beneficial effects for lycopene supplementation, at a dosage of 20–25 mg/day for six months in prostate cancer patients with high-grade prostatic intraepithelial neoplasia [81].

Clinical studies have reported an association for lycopene and other components of the Mediterranean diet with reduced occurrence and progression of colon cancer [134]. The RCT by Walfisch et al. studied in colon cancer patients who were candidates for colectomy the potential effects of oral lycopene integration in reducing plasma levels of insulin-like growth factor-I (IGF-I), a recognized risk factor for various cancers including colon cancer. The authors reported that lycopene extract taken with meals at a dosage of 30 mg twice daily for a variable period of time before surgery played a preventive role, reducing the plasma concentration of IGF-I by about 25% compared with the placebo group (*p* < 0.05) [80].

Several RCTs have examined the role of vitamin E in preventing neurotoxicity of chemotherapy regimens and relieving xerostomia and mucositis associated with radiotherapy.

Argyriou et al. and Pace et al. [83,85] showed that treatment with vitamin E (600 mg/day and 400 mg/day, respectively) in patients with solid tumors receiving cisplatin-based chemotherapy resulted in a reduction in the incidence of chemotherapy-induced peripheral neuropathy (CIPN) compared with patients who received placebo (*p* = 0.026 and *p* < 0.01, respectively). Argyriou et al. also showed that vitamin E (300 mg twice daily) protected cancer patients from the occurrence of paclitaxel-induced peripheral nerve damage [86].

A large study [87] conducted in patients with nonmyeloid solid tumors (n = 140) showed that recovery from CIPN was faster in the group of patients receiving taxane-based chemotherapy treated with vitamin E (400 mg twice daily) than in the control group (*p* = 0.01), suggesting that vitamin E might reduce the duration of CIPN.

Similarly, the studies by Afonseca et al. [88], Salehi et al. [89] and Kottschade et al. [90], finding no differences between the vitamin E-supplemented and placebo groups, questioned the benefits of this NHP supplementation in preventing CIPN.

The absence of a clear benefit, as in the study by Afonseca et al., could be due to the administration of vitamin E in combination with other substances (e.g., calcium and magnesium). This may have interfered with the vitamin’s antioxidant activity, making it less effective in preventing the peripheral neuropathy. Another concern relates to dosage; in fact, the daily dose of vitamin E used in the different RCTs ranged from 300 to 800 mg/day. The studies by Heiba et al. and Kottschade et al. [87,90], which enrolled the largest number of patients (n = 140 and n = 189, respectively), despite administering the highest dose, found no benefit.

Another form of neurotoxicity is ototoxicity, which is strongly associated with cisplatin-based chemotherapy. In this regard, it has been shown that vitamin E (400 mg/day) may also have a neuroprotective role, preventing hearing loss in patients with solid tumor treated with cisplatin-based chemotherapy compared with those in the control arm [92].

Vitamin E has also been evaluated for its efficacy in improving radiotherapy-induced oral symptoms such as xerostomia and mucositis. Ferreira et al. [91] demonstrated that rinsing the oral cavity in an oil solution containing vitamin E decreased the incidence of symptomatic oral radio-induced mucositis in patients with head and neck cancer. Chung et al. [105] reported that oral supplementation of vitamin E and C (100 IU vitamin E plus 500 mg vitamin C twice daily during radiotherapy) in patients with head and neck cancer improved xerostomia assessed at 1 month and 6 months after treatment (*p* = 0.008, and *p* = 0.007, respectively) compared with the control group.

Few RCTs, which are variable in cancer type, administration route and evaluated outcomes, have studied the potential benefits of vitamin C supplementation. The most important positive effects of vitamin C in terms of remission (*p* = 0.004) and overall survival (*p* = 0.039) in the supplemented group compared to the placebo group were found in the RCT of Zhao et al. [93], in which vitamin C was used intravenously at low doses in elderly patients with acute myeloid leukemia treated with decitabine-based chemotherapy.

Considering again hematologic cancers, in the study of van Gorkom et al. [94], patients with myeloma or lymphoma requiring autologous hematopoietic stem cell transplantation (HSCT), who are often vitamin C deficient, received intravenous vitamin C every 24 h until the day of discharge. After discharge, patients in the intervention group received oral vitamin C. Unfortunately, OS at 3 months, neutrophil recovery time, hospitalization time, and incidence of neutropenic fever did not differ between the intervention groups and placebo group.

Similarly, a large study (n = 442) [95] performed in patients with metastatic colorectal cancer (mCRC) treated with FOLFOX ± bevacizumab chemotherapy failed to show that high-dose vitamin C can increase Patient Free Survival (PFS) and OS compared with chemotherapy alone. However, the same study showed that supplementation can benefit patients with mCRC carrying the RAS mutation. Evaluation of adverse events according to CTCAE (v. 4.0) showed no clinically significant additional toxicity in the intervention arm compared with the placebo arm.

The study by Ma et al. [96] also evaluated the safety profile of high-dose intravenous vitamin C supplementation over a long follow-up of 5 years and in patients with stage III or IV ovarian cancer treated with carboplatin and paclitaxel. The study showed that vitamin C treatment did not increase the rate of grade 3 or 4 toxicities. However, mild-to-moderate grade toxicities were significantly less frequent in the intervention group than in the placebo group (*p* < 0.0001 and *p* = 0.003, respectively).

Oral administration of vitamin C in patients with cancer has been evaluated in RCTs that evaluated changes in various molecular factors. Mild protective effects in modulating inflammation have been reported in patients with esophageal adenocarcinoma undergoing radiotherapy, chemotherapy (5-fluorouracil and cisplatin) and surgery [98].

In addition, results of the study by Gilberg et al. [97] suggest that normalization of plasma vitamin C by oral supplementation in patients treated with 5-azacytidine may increase levels of the 5-methylcytosine (5 mC) and 5-hydroxymethylcytosine (5 hmC) ratio compared to patients treated with placebo, thereby enhancing the effects of 5-azacitidine.

Tumor type, route of administration, and dosage may influence the efficacy of vitamin C treatment. In van Gorkom’s study [94], for example, patients with myeloma or lymphoma in the active intervention group received intravenous vitamin C every 24 h until the day of discharge. However, after discharge, vitamin C was given orally.

In the large study by Wang et al. [95], however, patients with mCRC received high-dose intravenous vitamin C only for 3 days of each chemotherapy cycle, which may not be enough for vitamin C to exert its antitumor effect. Moreover, because supplementation was stopped at 6 months before most patients progressed, the impact of vitamin C on the tumor may be underestimated. In addition, the frequency of vitamin administration may also be important, as demonstrated in a mouse model by Campbell et al., who observed that the antitumor activity of ascorbate was greater after daily administration compared with infusions every other day [134].

Many patients take solutions containing vitamin mixes, the so-called multivitamins, with antioxidant activity during cancer therapy in order to improve outcomes or alleviate adverse effects of chemotherapy. RCTs that have evaluated the efficacy and safety profile of these supplements include the study of Bairati et al. [102], which investigated vitamin E and beta-carotene supplementation on head and neck cancer patients treated with radiation therapy. The supplements were administered during radiotherapy and for an additional 3 years. Contrary to expectations, cause-specific mortality rates tended to be higher in the intervention arm than in the placebo arm.

Similarly, Meyer et al. [103] tested the effects of oral supplementation of vitamin E and beta-carotene on the outcomes of smoking patients with head and neck cancer undergoing radiation therapy. Again, the intervention group demonstrated higher recurrence (*p* = 0.03), early mortality (*p* = 0.04) and all-cause mortality (*p* = 0.02) compared with the placebo group, leading to the hypothesis that the combined exposures of smoking and supplementation reduced the efficacy of radiotherapy.

The benefits of vitamin E and beta-carotene supplementation along with vitamin C were also evaluated in patients with stage IIIb and IV NSCLC treated with paclitaxel- and carboplatin-based chemotherapy, without finding significant differences in overall survival (OS) at one and two years (*p* =0.20) between the intervention and control arms [104].

Unlike previous studies, Cascinu et al. [106] measured cell proliferation in colon mucosa of patients with resected colorectal cancer using proliferating cell nuclear antigen (PCNA) after 6 months of oral supplementation with vitamin C, vitamin E, vitamin A and calcium.

The difference in the percentage of reduction of mean PCNA marking index (PCNALI) between baseline and 6 months after supplementation showed a decrease in both the intervention and placebo arms (*p* = 0.0001), suggesting that calcium and vitamin supplementation did not reduce the cellular kinetics of the colonic epithelium.

In conclusion, most RCTs have failed to demonstrate the real benefits of multivitamin supplementation in cancer patients; in fact, in several cases it has even been shown to be harmful. Among the few studies in favor of supplementation, the RCT by Suhail et al. [107] showed that co-administration of vitamins C and E in women with breast cancer restored antioxidant status (lowered by the presence of the tumor itself and chemotherapy) and reduced DNA damage, suggesting that these vitamins might be useful in protecting against chemotherapy-related side effects.

The effects of selenium supplementation (alone or in combination with vitamins) have been studied in patients with various malignancies.

Selenium is an essential mineral incorporated into selenoproteins, which, like vitamins, are involved in the antioxidant defense mechanisms. Furthermore, in the immune system, selenium stimulates the formation of antibodies and the activity of T helper cells, cytotoxic T cells and NK cells [58,135].

The antitumor activity of selenium has been mainly attributed to its ability to interfere with the synthesis of active redox proteins and, in general, to modulate cellular redox balance [136].

In the study of Rocha et al., an oral selenium supplementation for 60 days (at doses ranging from 27 to 100 mcg) alleviated treatment toxicities such as neutropenia and increased immunoglobulin A synthesis in patients with leukemia and solid tumors [109]. The combination of selenium and zinc in oral tablets administered for 50 days has also been shown to alleviate treatment-related asthenia and improve appetite in patients with gastrointestinal cancers [129]. Karp et al. investigated the incidence of second primary cancer in 1040 patients with Non-Small-Cell Lung Cancer (NSCLC) after a long follow-up of 7.9 years. No difference between patients who received 200 mcg daily of selenium or placebo was found [111]. In addition, a decrease in oxidative stress caused by iodine treatment, as measured by the detection of reduced concentrations of 8-epi-PGF2α, was evidenced after administration of 400 mcg selenium, combined with 2000 mg vitamin C, and 1000 mg vitamin E, given to patients for 21 days prior to therapy in patients with thyroid cancer [124].

Fuchs-Tarlovsky et al. evaluated the potential effects of an oral supplement containing selenium 15 mcg combined with beta-carotene 4.8 mg; vitamin C, 200 IU; and vitamin E, 200 IU, on hematological toxicity, QOL, and oxidative stress in patients with cervical cancer treated with radiotherapy and cisplatin-based chemotherapy. The authors found that NHP supplementation induced a trend toward reduced oxidative stress and maintained hemoglobin levels at a value of 12.50 ± 1.22 g/dL. In addition, QOL was significantly increased in the supplemented group compared to the placebo [123].

The study of Hopkins et al. evaluated the effects of 200 μg L-selenomethionine combined with several other antioxidant micronutrients, including 800 mg DL-α-tocopherol acetate, 24 mg β-carotene, 1.0 g vitamin C, 7.2 mg riboflavin, 80 mg niacin, 60 mg zinc, and 5 mg manganese administered daily for 4 months, in influencing levels of oxidative and inflammatory biomarkers in patients with a history of sporadic colorectal adenoma. Plasma concentration of TNF-α and cystine significantly decreased in the active treatment group by 37% (*p* = 0.002) and 19% (*p* = 0.03) compared with the placebo group. The decreases in TNF-α and cystine were more pronounced in nonsmokers [126].

## 7. Factors Influencing Therapeutic Outcomes of Patients Taking NHPs

One of the most important reasons why studies investigating the potential therapeutic role of NHPs are often inconsistent is the large number of variables that can influence clinical outcomes. Different study design and follow-up, enrolled patients’ characteristics, NHP dosing and formulation, concomitant treatments, and outcome measures are just some of them.

For example, with regard to the potential effects of vitamin D, the 25(OH)D level (< vs. >10 ng/dL) must be taken into account [137]. Morelli et al. conducted a very interesting study to assess the actual role of vitamin D in CCR, taking into account a number of potential predictors of deficiency in this micronutrient. First, they found that OS in patients with 25(OH)D > 10 ng/mL was longer compared to patients with lower levels (<10 ng/mL). Subsequently, when examining other variables, including hematological parameters, the neutrophil-to-lymphocyte ratio (NLR) was found to be the most powerful predictor of vitamin D deficiency. When NLR and 25(OH)D levels were considered simultaneously, patients with NLR < 3.5 plus 25(OH)D > 10 ng/mL had a longer OS than NLR > 3.5 plus 25(OH)D < 10 ng/mL (*p* = 0.0004) [137].

Several clinical studies have examined how dietary vitamin D intake affects the intestinal bacterial composition resulting in anti-inflammatory potential [138,139], and the role of vitamin D on the modulation of the gut microbiota; consequently, the possible effect of supplementation in patients with CRC have been extensively investigated [139,140,141,142]. Women seem to have lower vitamin D absorption than men, due to a different metabolism and the presence of hormones influencing different bacterial species in the microbiota [143]. A randomized phase II clinical trial showed that CRC patients who took 2000 IU of vitamin D daily for one year experienced a change in their gut microbiota and amino acid biosynthetic pathways. Although this study was conducted on only 60 patients, it showed that women (n. 30/60) taking the supplement had lower vitamin D levels at baseline than men and that supplementation closed this gap [100]. Sex/gender may also influence the effects of other vitamins. Mooney et al. examined the potential of vitamin supplementation [500 mg vitamin C and 400 IU vitamin E per day] in men and women who smoke at least 10 cigarettes per day in reducing benzo(a)pyrene [B(a)P] adducts, a well-established marker of cancer risk [144]. No treatment benefits were found in the entire study population and among men, whereas in women B(a)P-DNA adducts decreased by 31% compared to placebo (*p* = 0.03).

Hercberg et al. performed an RCT within the framework of ‘The Supplementation in Vitamins and Mineral Antioxidants’ (SU.VI.MAX) study to evaluate the effect of daily supplementation of vitamins and minerals (120 mg of vitamin C, 30 mg of vitamin E, 6 mg of β-carotene, 100 μg of selenium and 20 mg of zinc) in reducing the risk of skin cancer (SC) in a large sample of volunteers (7876 women and 5141 men) followed for 7.5 years. No difference was found in SC frequency among men, whereas the incidence of SC (including melanoma) in women was higher in women who received supplementation (HR= 1.68; *p* = 0.03) compared to placebo [145]. Overall, these results suggest that further clinical investigations, with a larger number of patients and appropriate stratification according to several covariates, including sex/gender, are needed to clarify the potential beneficial effect of vitamin supplementation against cancer.

Another issue is ascribed to the specific properties of NHPs, but also to epigenetic modifications possibly induced. A phase 3 RCT, the ‘Oral Nicotinamide to Reduce Actinic Cancer’ (ONTRAC), demonstrated that 500 mg nicotinamide (NAM) twice daily is safe and effective in preventing nonmelanoma SC and reducing the number of actinic keratosis in high-risk patients. Notably, while the number of actinic keratosis was reduced as early as 3 months and further at 6, 9 and 12 months, positive results regarding cancer lesions were found after a 12-month treatment but not after a shorter period. These beneficial effects were lost after NAM discontinuation [146].

NAM, as a component of ARCON (carbogen + NAM), has been also recognized as valuable treatment for head and neck, bladder [147] and laryngeal neoplasms [148]. Compared with accelerated radiotherapy (AR) only, ARCON reduced local relapse rate and improved bladder conservation rate and OS in patients with stage II-to-IV laryngeal cancer. However, such benefits were found only in hypoxic tumors and not in those which were well oxygenated [148].

Hypoxic changes are strongly correlated with changes in microenvironment and tumor metabolism, including modulation of the oxidative state and, more properly, redox homeostasis. This is another very important aspect, since ROS, when produced in excess of the antioxidant molecules, induce dysfunction of molecular mechanisms crucial for cellular defense and maintenance of redox balance, causing chronic oxidative stress and inflammation, which characterize age-associated diseases [149,150,151,152], and promote DNA damage and carcinogenesis [153].

Indeed, NHPs could exert anticancer effects mainly due to their antioxidant and anti-inflammatory characteristics and their ability to stimulate the activation of enzymes essential for the regulation of oxidative and inflammatory homeostasis [154], also helping to overcome the effects of chemotherapy-induced oxidative stress in cancer patients [155].

Given the wide use of NHPs in the general population and in cancer patients [156], an important issue currently being debated is the inter-individual variability in the magnitude of effects (both beneficial and adverse) due to NHP– drug interactions.

This aspect is crucial, considering that a significant proportion of cancer patients take NHPs during cancer treatment without the knowledge of their physicians [157,158] and supplement–drug interactions may led to adverse events associated to pharmacokinetic and pharmacodynamic interactions due to a possible alteration of the efficacy and safety profiles of anticancer drugs [158].

## 8. Conclusions

Some RCTs studying the effects of silymarin (topical and oral), ginsenoside (oral), and oral vitamin E (alone or combined with other NHPs) might be useful against adverse events related to radiotherapy or chemotherapy with capecitabine or treatment with doxorubicin alone or combined with paclitaxel and cyclophosphamide. Curcumin (topical and oral) and lycopene (oral) could provide some benefit in patients with prostate cancer by reducing PSA levels.

Overall, the results of available RCTs do not allow significant conclusions to be drawn about the role of NHPs in cancer. Notably, there are conflicting results among in vitro studies that show clear beneficial effects of NHPs in influencing inflammatory and oxidative status, survival, and proliferation of cancer cells and RCTs that, in contrast, have often found little or no benefit for cancer patient survival and therapy-related adverse effects. Moreover, although NHPs have relatively low toxicity, their wide use among cancer patients, often without the knowledge of treating physicians, also creates concern. Further large RCTs are needed to determine precise indications and the correct route and timing of administration before considering the addition of NHPs to clinical practice.

## Figures and Tables

**Table 1:** Characteristics and main results of RCTs available in the literature over the past two decades that have tested the effects of herbal supplements in different types of cancer.

References (Years)	Placebo	Country	Patients (*n*)	Sex (F%)	Cancer	NHPs (Dosage)	NHPs (Timing of Administration)	Follow-Up	Outcomes	Results and Conclusions
Resveratrol										
Howells et al. [62] (2011)	Yes	Europe	9 INT-g: 6 PL-g: 3	INT-g: 16.6% PL-g: 66.7%	Stage IV colorectal cancer and hepatic metastases	ORALLY SRT501 (5 g/day)	Up to a minimum of 10 and a maximum of 21 days.	NA	Safety and tolerability of SRT501 and resveratrol pharmacokinetics.	Nausea and mild diarrhea were recorded with administration of SRT501. Other adverse events such as chills, lethargy, rash, skin irritation, and vascular redness were resolved without consequences.
Genistein										
Lazarevic et al. [63] (2011)	Yes	Europe	47: INT-g: 23 PL-g: 24	0%	Prostate cancer	ORALLY Genistein (30 mg/day)	3–6 weeks before radical prostatectomy.	NA	Modulation of PSA levels in serum and prostatic tissue and serum testosterone.	This study shows a possible effect of genistein in the treatment of early prostate cancer, producing a significant reduction in serum PSA and a stabilization of PSA expression in malignant prostate tissue.
Ginsenoside										
Hamidian et al. [64] (2023)	Yes	Europe	30 INT-g: 15 PL-g: 15	100%	Non-metastatic breast cancer	ORALLY Ginseng (1 g/day)	NA	At baseline, after the fourth and eighth CT cycles.	Influence of ginseng on the prevention of cardiac dysfunction related to doxorubicin-based therapy.	Ginseng may protect against doxorubicin-induced cardiac dysfunction.
Yennurajalingam et al. [65] (2017)	Yes	Asia	112 INT-g: 56 PL-g: 56	NA	Breast, gastrointestinal, genitourinary, gynecologic, thoracic and other advanced cancers	ORALLY Panax ginseng (400 mg/twice a day)	28 days	NA	Effect on cancer-related fatigue.	After 4 weeks of treatment, patients treated with supplementation did not appear to have obtained any positive effects compared with the placebo group.
Omrani et al. [66] (2023)	Yes	Asia	40 PG1-g: 10 PG2-g: 10 CTR-g: 10 PL-g: 10		Thyroid cancer	ORALLY Panax ginseng (500 mg/day)	2 days before or 2 days before to 1 day after radioiodine therapy.	NA	Efficacy for genotoxicity by radioiodine therapy.	Panax ginseng seems to be able to protect thyroid cancer by genotoxicity related to radioiodine.
Silymarin										
Moezian et al. [67] (2022)	Yes	Europe	30 INT-g: 15 PL-g: 15	100%	Non-metastatic breast cancer	ORALLY Silymarin (140 mg/3 times a day)	1 month	At 1 month	CT-induced hepatotoxicity in patients who received doxorubicin/cyclophosphamide-paclitaxel regimen.	Silymarin could significantly reduce hepatotoxicity severity after 1 month of treatment.
Karbasforooshan et al. [68] (2018)	Yes	Asia	40 INT-g: 20 PL-g: 20	100%	Breast cancer	TOPICAL Silymarin (at least 30 g in 3 weeks)	5 weeks	5 weeks	Radiodermatitis.	The use of 1% silymarin gel once a day can reduce the severity of radiodermatitis and delay its onset.
Elyasi et al. [69] (2016)	Yes	Asia	30 INT-g: 15 PL-g: 15	52%	Head and neck cancer	ORALLY Silymarin (420 mg/day)	6 weeks	6 weeks	Mucositis induced by radiotherapy.	Oral administration of silymarin at daily dose of 420 mg seems to be able to reduce the severity grade of radiotherapy-related oral mucositis and delay its occurrence.
Elyasi et al. [70] (2017)	Yes	Asia	40 INT-g: 20 PL-g: 20	Silymarin-g: 45% PL-g: 35%	Gastrointestinal cancer	TOPICAL Silymarin (1%, 2 times a day)	9 weeks	9 weeks	Capecitabine-induced Hand–Foot Syndrome.	The application of silymarin gel 1% twice a day may reduce the severity of capecitabine-induced Hand–Foot syndrome and delay its occurrence.
Shahbazi et al. [71] (2015)	Yes	Asia	30 INT-g: 15 PL-g: 15	Silymarin-g: 25% PL-g: 50%	Gastrointestinal, ovarian cancer, mesothelioma	ORALLY Silymarin (420 mg/day in three divided doses)	From 24 to 48 h before the initiation of cisplatin infusion to the end of three 21-day cisplatin-containing CTs.		Cisplatin nephrotoxicity.	Conventional form of silymarin tablets at a daily dose of 420 is not effective against cisplatin nephrotoxicity.
Curcumin										
Howells et al. [72] (2019)	No	Europe	27 FOLFOX-g: 9 FOLFOX + curcumin-g: 18	NA	Metastatic colorectal cancer	ORALLY Curcumin (2 g/day)	NA	NA	Safety and efficacy of curcumin in patients receiving folinic acid/5- fluorouracil/oxaliplatin chemotherapy.	Curcumin is a safe and tolerable adjunct to FOLFOX.
Shah et al. [73] (2020)	NA	NA	NA	NA	Head and neck cancer	OROPHARYNGEAL USE Curcumin 0.1%	NA	NA	Compare the effectiveness and safety of 0.1% curcumin and 0.15% benzydamine mouthwash on RIOM.	The mouthwashes examined were not able to prevent the onset of RIOM and reduce the severity of RIOM; the use of 0.1% curcumin mouthwash was able to significantly delay the onset of RIOM.
Talakesh et al. [74] (2022)	Yes	Asia	42	NA	Breast cancer	ORALLY Nano-curcumin capsules (80 mg/day	NA	NA	Radiation-induced skin reactions.	The administration of nano-curcumin should be able to alleviate radiation- induced skin toxicity, but this effect was not significant.
Choi et al. [75] (2019)	Yes	Asia	97 INT-g: 49 PL-g: 48	0%	Prostate cancer patients who received IAD	ORALLY Curcumin (1440 mg/day)	6 months	Until the beginning of the second on-treatment.	Anti-cancer activity.	Intake of oral curcumin did not significantly affect the overall off-treatment duration of IAD. PSA elevation was suppressed with curcumin intake.
Chaiworramukkul et al. [76] (2022)	Yes	Asia	33 INT-g: 17 PL-g: 16	INT-g: 29.4% PL-g: 12.5%	Solid malignancy	ORALLY Curcumin (800 mg/twice a day)	8 weeks	8 weeks	Prevention of CACS.	Curcumin was not shown to be superior to placebo.
Ryan et al. [77] (2013)	Yes	Europe	30 INT-g: 14 PL-g: 6	100%	Breast cancer	ORALLY Curcumin (6 g/day)	During RT.	Weekly, after every fifth RT session, at the end of RT, and at two post-RT appointments (1 month and 6 months post-RT.	Radiation dermatitis severity.	6 g of curcumin during radiotherapy can reduce the severity of radiation-related dermatitis.
Hejazi et al. [78] (2016)	Yes	Asia	40 INT-g: 20 PL-g: 20	0%	Prostate cancer	ORALLY Curcumin (3 g/day)	During RT.	NA	Oxidative status.	Curcumin can increase the total antioxidant capacity without compromising the efficacy of RT.
						Lycopene				
Kucuk et al. [79] (2002)	No	America	26 INT-g: 15 CTR-g: 11	NA	Prostate cancer	ORALLY Lycopene (30 mg/day)	For 3 weeks before radical prostatectomy.	NA	Effects of lycopene supplementation on the prostate tissues and on serum levels of PSA, IGF-l, and IGFBP-3.	Mean plasma levels of PSA were lower in the intervention group than in the control group. Based on this evidence, lycopene could have beneficial effects in prostate cancer.
Walfisch et al. [80] (2007)	Yes	Asia	56 INT-g: 30 PLC-g: 26	37.5%	Colon cancer	ORALLY Lycopene extract (30 mg/twice a day)	Variable period of time before surgery (until 65 days).	NA	Comparing concentrations of Insulin Like Growth Factor-I and its binding protein	Since high plasma levels of insulin-like growth factor-I are a risk factor, tomato lycopene extract has a preventive role against colon and possibly other cancers.
Mariani et al. [81] (2014)	No	Europe	32	NA	High-grade prostatic intraepithelial neoplasia, prostatitis and prostate cancer	ORALLY Lycopene (20–25 mg/day)	6 months	NA	Plasma and prostate concentration of lycopene after a lycopene-enriched diet and their relevance in tumor progression	This study suggest a connection between low prostate lycopene concentrations and prostate cancer
Clark et al. [82] (2006)	No	America	36	0%	Prostate cancer	ORALLY Lycopene (15, 30, 45, 60, 90, and 120 mg/day)	1 year	NA	Efficacy, pharmacokinetics, and toxicity/tolerability of lycopene	Lycopene supplementation in patients with prostate cancer is safe and well tolerated. Lycopene supplementation at 15 to 90 mg/day did not result in any discernible response in serum PSA.

Abbreviations: Micronized resveratrol, SRT501; Radiotherapy, RT; Chemotherapy, CT; Leucovorin calcium (folinic acid), fluorouracil, and oxaliplatin, FOLFOX; Radiation-induced oral mucositis, RIOM; Intermittent androgen deprivation, IAD; Prostate-specific antigen, PSA; Cancer anorexia–cachexia syndrome, CACS; Insulin-like growth factor-1, IGF-1.

**Table 2:** Characteristics and main results of RCTs available in the literature over the past two decades that have tested the effects of vitamins and other micronutrients in different types of cancer.

References (Years)	Placebo	Country	Patients (n)	Sex (F%)	Cancer	NHPs (Dosage)	NHPs (Timing of Administration)	Follow-Up	Outcomes	Main Results
Vitamin E										
Argyriou (a) [83] (2006)	yes	Europe	35 INT-g: 16 PL-g: 19	INT-g: 31.3% PL-g: 31.6%	Lung, cervix, testicular, head and neck and gastric cancer treated with cisplatin	ORALLY vitamin E (600 mg/day)	During CT and 3 months after its cessation.	3 months after CT.	CIPN	Vitamin E protects cancer patients from the occurrence of CIPN effectively and safely.
Pace (a) [84] (2003)	yes	Europe	27 INT-g: 13 PL-g: 14	NA	Lung, ovarian rhinopharynx, urethral gastric, testicular, esophageal, ethmoidal, and tongue cancer	ORALLY vitamin E (300 mg/day)	Before CT and 3 months after the suspension of cisplatin.	3 months after cisplatin.	(1) TNS (2) severity of neurotoxicity	The incidence of neurotoxicity was significantly lower in INT-g than in PL-g. The severity of neurotoxicity was lower in patients supplemented with vitamin E.
Pace (b) [85] (2010)	yes	Europe	41 INT-g: 17 PL-g: 24	INT-g: 47.7% PL-g: 41.7%	Patients with solid malignancies treated with cisplatin	ORALLY vitamin E (400 mg/day)	Before CT and 3 months after the suspension of cisplatin.	3 months after cisplatin.	(1) TNS (2) severity of neurotoxicity	The incidence of neurotoxicity was significantly lower in INT-g than in PL-g. The severity of neurotoxicity was significantly lower in INT-g than in those receiving placebo.
Argyriou (b) [86] (2006)	yes	Europe	32 INT-g: 16 PL-g: 16	INT.g: 55.6% PL-g: 68.4%	Lung, breast and ovarian cancer treated with paclitaxel	ORALLY vitamin E (300 mg twice a day)	During CT and 3 months after its cessation.	3 months after CT.	PIPN	Neurotoxicity occurred in 18.7% of INT-g and 62.5% of PL-g (*p* = 0.03). The RR of developing PIPN was significantly higher in the PL-g.
Heiba et al. [87] (2021)	yes	Africa	140 INT-g: 70 PL-g:70	INT-g: 92.9% PL-g: 95.7%	Breast (70%), cervical, endometrial, gastric, NSCLC, ovarian, prostate cancer treated with Taxane	ORALLY vitamin E (400 mg twice daily)	Starting with CT and for 1 month after its completion.	1 months after CT.	(1) Incidence of grade ≥ 2 neuropathy (CTCAE v 5.0) (2) time to onset and duration of grade ≥ 2 neuropathy.	Vitamin E has no protective role in reducing the incidence of taxane-induced neuropathy. However, vitamin E could reduce the duration and severity of CIPN.
Afonseca et al. [88] (2013)	yes	Africa	34 INT-g: 18 PL-g: 16	INT-g: 44.4% PL-g: 50%	CRC and gastric cancer treated with oxaliplatin	ORALLY vitamin E (400 mg/day)	Until after the end of the oxaliplatin-based CT regimen.	End of the CT regimen.	PNP (CTCAE v.3)	The use of vitamin E did not demonstrate a significant reduction in the incidence of OIPN in patients with colorectal and gastric cancer treated with oxaliplatin.
Salehi et al. [89] (2015)	yes	Asia	65 INT-g: 32 PL-g: 33	INT-g: 25% PL-g: 48.5%	CRC treated with oxaliplatin	ORALLY vitamin E (400 mg/day)	Until after the sixth course of the oxaliplatin-based CT regimen.	After the sixth course of CT completion.	PNP	Lack of benefit of vitamin E in preventing OIPN in patients with colorectal and gastric cancer treated with oxaliplatin.
Kottschade et al. [90] (2011)	yes	America	189 INT-g: 96 PL-g: 93	INT-g: 83% PL-g: 81%	Breast, lung and other cancer treated with taxanes (109), cisplatin (8), carboplatin (2), oxaliplatin (50), or combination (20)	ORALLY vitamin E (400 mg/2 day)	Within 4 days of the first CT and continued for 1 month beyond completion of CT.	1 month after completion of CT.	Incidence of grade 2+ SN (CTCAE v 3.0)	No difference in the incidence of grade 2+ SN between the two groups. There were no significant differences between treatment arms for time-to-onset of neuropathy and CT dose reductions due to neuropathy.
Ferreira et al. [91] (2004)	yes	Africa	54 INT-g: 28 PL-g: 26	INT-g: 10.7% PL-g: 11.5%	HNC treated with RT	RINSING OF THE ORAL CAVITY with OIL SOLUTION containing vitamin E (400 mg) before and after RT	During the 5-to-7 weeks of RT.	Fifth to seventh week of RT.	Severity of oral mucositis.	Symptomatic mucositis was observed significantly less in INT-g than in PL-g. Vitamin E reduced the risk by 36% and reduced grade 2 to grade 3 pain during RT.
Villani et al. [92] (2016)	yes	Europe	23 INT-g: 13 PL-g: 10	NA	Patients with solid malignancies treated with cisplatin	ORALLY vitamin E (400 mg/day)	Starting with CT and for 3 month after the discontinuation of cisplatin.	1, 2, and 3 months.	Cisplatin-induced ototoxicity. (Audiograms and evoked brainstem responses.)	At 1 month, a significant hearing loss in PL-g at both 2000 HZ (right ear: *p* = 0.05; left ear: *p* = 0.04) and 8000 HZ (right ear: *p* = 0.04; left ear: *p* = 0.03) was detected when compared to baseline values.
Vitamin C										
Zhao et al. [93] (2018)	yes	Asia	73 INT-g: 39 PL-g: 34	INT-g: 46% PL-g: 44%	AML in elderly patients treated with DCAG chemotherapy	INTRAVENOUS vitamin C (50–80 mg/kg/day)	Until relapse or progressive disease, death, or unacceptable toxicity occurred.	14 months	Clinical outcomes: - CR - OS	Vitamin C (IV) at low doses of DCAG appeared to improve CR and prolong OS, compared with DCAG alone, in elderly patients with AML.
van Gorkom et al. [94] (2022)	yes	Europe	44 INT-g: 21 PL-g: 23	INT-g: 57% PL-g: 57%	Myeloma or Lymphoma	vitamin C INTRAVENOUS (70 mg/Kg/day) ORALLY (500 mg/2 day)	Until day 42 of the study.	3 months	- time to neutrophil recovery - hospitalization time - OS	Neutrophil recovery time, hospitalization time, incidence of neutropenic fever and OS at 3 months did not differ between the two groups.
Wang et al. [95] (2022)	yes	Asia	442 INT-g: 221 PL-g: 221	INT-g: 39.8% PL-g: 37.6%	mCRC treated with FOLFOX ± bevacizumab	INTRAVENOUS high-dose vitamin C (1.5 g/kg/d, for 3 h from D1 to D3)	12 treatment cycles.	Survival: every 9 weeks; adverse events at 30 or 90 days after treatment end.	- PFS - ORR - OS - adverse events (CTCAE V.4)	High-dose vitamin C during CT failed to show superior PFS and OS compared with CT alone in patients with mCRC but may be beneficial in patients with mCRC with RAS mutation. Vitamin C supplementation resulted in no clinically significant additional toxicity compared with treatment with CT alone.
Ma et al. [96] (2014)	yes	America	25 INT-g: 13 PL-g: 12	NA	Stage III or IV ovarian cancer treated with carboplatin and paclitaxel	INTRAVENOUS vitamin C dose escalation initiated at 15 g, titrated up to a therapeutic range of 75 or 100 g (two times per week)	In conjunction with CT for 6 months, and for another 6 months after CT completion.	5 years	Safety of high-dose intravenous Vit. C by CTCAE v3.	Vitamin C treatment did not increase the rate of grade 3 or 4 toxicity. Moreover, grade 1 and 2 toxicities were significantly less in the INT-g than in the PL-g.
Gillberg et al. [97] (2019)	yes	Europe	20 INT-g: 10 PL-g: 10	INT-g: 10% PL-g: 50%	Myeloid cancers treated with 5-azacytidine	ORALLY vitamin C (500 mg/day)	The last 2 cycles of CT.	2 cycles of CT	Vitamin C deficiency and 5-methylcytosine (5 mC)- to-5-hydroxymethylcytosine (5 hmC) ratio.	Normalization of vitamin C levels by oral supplementation results in increased 5 hmC/5 mC ratio compared with the placebo arm and may potentiate the biological effects of 5-azacytidine.
Abdel-Latif et al. [98] (2018)	yes	Europe	20 INT-g: 9 PL-g: 11	20%	Esophageal adenocarcinoma undergoing RT, CT (5-fluorouracil and cisplatin) and surgery	ORALLY vitamin C (1000 mg/day)	4 weeks	4 weeks	Pre- and post-Vit. C endoscopic biopsies were used for NF-κB activity and cytokine analysis.	There was a significant reduction in cytokines levels in INT-g.
Vitamin D										
Vahedpoor et al. [99] (2018)	yes	Asia	58: Tot 29: Vit. C-g 29: Cg	100%	CIN2/3	ORALLY vitamin D3 (50,000 IU every 2 weeks)	for 6 months	6 months	Recurrence and metabolic status of patients of grade 2 or 3 CIN.	Vitamin D3 supplementation for 6 months had beneficial effects on CIN1/2/3 recurrence; however, it did not affect CIN2/3 recurrence.
Bellerba et al. [100] (2022)	yes	Europe	60: Tot 28: Vit. D-g 32: Cg	NA	CRC	ORALLY vitamin D3 (2000 IU/day)	1 year	1 year	- taxa/pathways - 25(OH)D levels.	Vitamin D supplementation may influence the gut microbiota, and the microbiota may partially mediate the effect of supplementation on 25(OH)D. Sex differences with respect to Vit. D levels have been found in the microbiota.
Urashima et al. [101] (2019)	yes	Asia	417: Tot 251: Vit. D-g 166: Cg	31%: Vit. D-g 38%: Cg	Digestive Tract Cancers	ORALLY vitamin D3 (2000 IU/day)	NA	Every month for the first 6 months, every 2 months for the second 6 months, and every 3 months thereafter until 5 years.	- survival	Vitamin D supplementation, compared with placebo, did not result in significant improvement in relapse-free survival at 5 years in patients with digestive tract cancer.
Multivitamins										
Bairati et al. [102] (2006)	yes	North America	540 Vit. E/ /b-carotene or Vit. E alone: 273 PL-g: 267	INT-g: 18% PL-g: 24%	HNC treated by RT	ORALLY vitamin E (400 IU/day) b-carotene (30 mg/day) After the first 156 patients the trial was continued with vitamin E alone.	During RT and for 3 additional years.	6 months during the 3 years following the end of RT and then once a year until the end of the study.	All-cause and cause-specific mortality rates.	All-cause mortality was significantly increased in INT-g. Cause-specific mortality rates tended to be higher in the supplement arm than in the PL arm. High-dose vitamin E could be harmful.
Mejer et al. [103] (2008)	yes	North America	540 Vit. E/ /b-carotene or Vit. E alone: 273 PL-g: 267	INT-g: 18% PL-g: 24%	HNC (stage I or II) in smoker patients treated by RT	ORALLY vitamin E (400 IU/day) b-carotene (30 mg/day)	During RT and for 3 years thereafter	3 years following the end of radiation therapy and then once a year.	- SPC - cancer-free survival - acute adverse effects of RT - recurrence	Combined exposure of cigarette smoking and vitamin supplementation reduced the efficacy of radiotherapy in terms of HNC recurrence, early HNC mortality and all-cause mortality.
Pathak et al. [104] (2005)	yes	Asia	136 INT-g: 64 PL-g: 72	NA	NSCLC (stage IIIb and IV) treated with CT (paclitaxel and carboplatin)	ORALLY vitamin E (1050 mg/day) vitamin C (6100 mg/day) b-carotene (60 mg/day)	NA	NA	- RR - median survival times - OS	RR, median survival times and OS rates showed no significant differences between the control arm and the experimental arm with vitamin supplementation.
Chung et al. [105] (2016)	yes	Asia	45 INT-g: 25 PL-g: 20	INT-g: 12% PL-g: 10%	HNC treated with RT	ORALLY vitamin E (100 IU) vitamin C (500 mg/2 day)	During RT	6 months	Xerostomia severity	INT-g showed greater improvements in xerostomia questionnaire score at 6 months post-RT. Short-term supplementation with an antioxidant vitamin E/C complex exerts a protective effect against RT-induced xerostomia.
Cascinu et al. [106] (2000)	yes	Europe	77 INT-g: 33 PL-g: 43	INT-g: 35.5% PL-g: 53.3%	Resected CRC	ORALLY vitamin C (1 g/day) vitamin E (70 mg/day) vitamin A (30,000 IU/day) natural culcium (2 g/day).	6 months	At the time of surgery and after 6 and 12 months of treatment.	Cell proliferation in the colonic mucosa using PCNA.	Calcium and vitamin supplementation does not reduce cell kinetics of colon epithelium.
Suhail et al. [107] (2010)	yes	Asia	40 INT-g: 20 PL-g: 20	100%	Breast cancer (stage II) treated with 5-fluorouracil, doxorubicin and cyclophosphamide	ORALLY vitamin E (400 mg/day) vitamin C (500 mg/day)	During and for 3 weeks after CT cessation.	3 weeks after CT cessation.	Activity of superoxide dismutase, catalase, glu- tathione-S-transferase and glutathione reductase and levels of malondialdehyde.	Co-administration of vitamin C and E in women with breast cancer restored antioxidant status (lowered by the presence of the tumor itself and chemotherapy) and reduced DNA damage, suggesting that these vitamins might be useful in protecting against chemotherapy-related side effects.
van Zandwijk et al. [108] (2000)	yes	Europe	2573 Retynil-g: 647 NAC-g: 642 Retynil/NAC-g: 643 PL-g: 641	13%	HNC and lung cancer	vitamin A- (retinyl) (300,000 IU and 150,000 IU daily) N- Acetylcysteine (NAC) (600 mg daily)	For 2 years	2 years	Recurrence, second primary tumor, death.	Survival, event-free survival, or second primary tumors after 2-year supplementation of retinyl palmitate and/or NAC resulted in no benefit for patients with head and neck cancer or with lung cancer.
Selenium										
Rocha et al. [109] (2016)	Yes	Latin America	36 INT-g: NA PL-g: NA	36%	ST: Bone, central nervous system, rhabdomyosarcoma, histiocytosis, kidney/gastrointestinal. LL: Hodgkin’s lymphoma, acute lymphoblastic and myeloid leukemia	ORALLY Selenium (27, 36, 54, 72, 100 mcg/day)	60 days	NA	Hematological cellular count; immunoglobulin synthesis (IgA, IgE, IgM, and IgG).	Selenium supplementation in patients with TS or LL was associated with increased neutrophil counts. After supplementation, IgA and IgG were higher in patients with ST than in those with LL. Selenium supplementation could alleviate cases of neutropenia.
Son et al. [110] (2017)	Yes	Asia	16 INT-g: 8 PL-g: 8	62.5%	Papillary thyroid cancer	ORALLY Selenium (300 mcg/day)	10 days (from 3 days before to 6 days after I treatment).	6 months after 131I treatment.	Radiation damage of the salivary Glands.	Selenium supplementation during 131I treatment was effective in reducing radiation damage of the salivary glands.
Karp et al. [111] (2013)	Yes	America	1561 INT-g: 1040 PL-g: 541	51.3%	NSCLC	ORALLY Selenium (200 mcg/day)	48 months	7.9 years	Incidence of second primary tumors.	Selenium was safe but not better than placebo in the prevention of second primary tumors in patients with resected NSCLC.
Laali et al. [112] (2019)	Yes	Asia	71 INT-g: 37 PL-g: 34	29.5%	HNC	ORALLY Selenium (200 mcg/twice a day)	Until the last day of RT.	Weekly	Effects on oral mucositis.	Selenium supplementation does not appear to affect the severity and duration of oral mucositis.
Dehghani et al. [113] (2023)	No	Asia	30 INT-g: 16 PL-g: 14	60%	Diffuse Large B-Cell Lymphoma	ORALLY Selenium (200 mcg/day)	3 months	3 months	Profile of CD4+ T-helper subsets including IFN-γ+/IL-4- Th1, IFN-γ-/IL-4+ Th2, and CD4+IL-17+ Th17 cells.	Selenium supplementation could reduce the proportion of CD4+ IL-17+ Th17 cells in diffuse large B-cell lymphoma patients at stable remission phase.
Muecke et al. [114] (2010)	Yes	Europe	81 INT-g: 39 PL-g: 42	100%	Cervical and uterine cancer treated with RT	ORALLY Selenium (500 mcg on the days of RT decreased to 300 μg on the days without treatment)	Until the last day of RT.	Until the last day of RT.	Potential relation between PTV of the RT and radiation-induced diarrhea.	The incidence of grade-2 or higher diarrhea was lower in the group taking selenium than in the control group. Selenium supplementation during RT could reduce the incidence and severity of diarrhea, especially in patients with large PTV (>1302 mL).
Vieira et al. [115] (2015)	Yes	Latin America	39	NA	Bone, CNS, AML, acute lymphocytic leukemia, Rhabdomyosarcoma, Hodgkin lymphoma, histiocytosis, kidney and genitourinary tract cancers	ORALLY Selenium (27, 36, 54, 72, and 100 mcg/day depending by age)	60 days	1 year	Quality of life of oncologic patients; valuation of AST, ALT, creatinine, and urea.	Supplementation with Selenium promotes the reduction in chemotherapy side effects in cancer patients, especially of fatigue, nausea, and impaired physical function. Renal and liver functions have also improved.
Karamali et al. [116] (2015)	Yes	Asia	58 INT-g: 28 PL-g: 28	100%	CIN1	ORALLY Selenium (200 mcg/day)	6 months	6 months	Regression and metabolic status of patients.	A greater percentage of women in the Selenium group had regressed CIN1 (*p* = 0.01) compared with those in the placebo group.
Duffield-Lillico et al. [117] (2003)	Yes	America	1312	NA	Patients with a history of two or more basal cell carcinomas or one or more squamous cell carcinomas of the skin	ORALLY Selenium (200 mcg/day)	3 years	10 years	Preventing nonmelanoma skin cancer.	Selenium supplementation is ineffective at preventing nonmelanoma skin cancer.
Goossens et al. [118] (2016)	Yes	Europe	292 INT-g: 151 PL-g: 141	0%	Bladder cancer	ORALLY Selenium (200 μg/day)	43 months	2 years	Chemoprevention of recurrence of bladder cancer.	Selenium is unable to reduce the likelihood of recurrence in patients with bladder cancer.
Muecke et al. [119] (2014)	Yes	Europe	81 INT-g: 39 PL-g: 42	100%	Cervical and uterine cancer	ORALLY Selenium on the days RT (500 µg/day) and selenium without treatment until the last day of RT (300 µg/day)	Until the last day of RT.	70 months after the end of RT.	Long-term survival.	Selenium intake did not affect the effectiveness of radiotherapy against cancer. It also did not adversely affect the long-term survival of patients.
Dziaman et al. [120] (2009)	Yes	Europe	260 INT-g: 70 PL-g: 202	100%	Breast and ovarian cancer	ORALLY Selenium (300 μg/day)	NA	NA	Oxidative stress/DNA damage.	Selenium supplementation results in reduction of oxidative DNA damage.
Bryan et al. [121] (2023)	Yes	Europe	270 Selenium-g: 65 Vit. E-g: 71 Selenium+ Vit. E-g: 69 PL-g: 65	25%	NMIBC	ORALLY Selenium (200 μg/day) vitamin E (200 IU/day)	1.5 years	39 months	Recurrence-free interval (RFI).	Administration of selenium was not correlated with a decreased risk of NMIBC recurrence. The use of vitamin E was associated with a significantly increased risk of recurrence.
Hanson et al. [122] (2007)	No	Europe	13 INT-g: 13	NA	CRC	ORALLY Selenium (60 mcg/die) vitamin E (700 mg/die) vitamin C (90 mg/die)	2 weeks	2 weeks	Activity of NK cells.	Vitamin E and selenium could enhance NK cell function in patients with colorectal cancer.
Fuchs-Tarlovsky et al. [123] (2013)	Yes	Latin America	103 INT-g: 49 PL-g: 54	100%	Cervical cancer	ORALLY Selenium (15 mcg/day) vitamin E (200 IU/day) vitamin C (200 IU/day) β-carotene (4.8 mg/day)	6 weeks	NA	Hematological toxicity, QOL, and oxidative stress.	There was a significant increase in protein oxidation level in those patients that were not receiving NHP supplement (*p* < 0.05). In the supplemented group serum hemoglobin was 12.50 ± 1.22 g/dL, while it decreased to 11.62 ± 1.36 g/dL (*p* < 0.05) in the placebo group. QOL was higher in the supplemented group when compared with the placebo group (*p* < 0.025).
Rosário et al. [124] (2016)	No	Latin America	40 INT-g: 20 CTR-g: 20	82.5%	Thyroid cancer	ORALLY Selenium (400 mcg/day) vitamin E (1000 mg/day) Vitamin C (2000 mg/day)	21 days before 131I.	2 and 7 days after 131I.	Oxidative stress caused by Iodine treatment.	The oxidative stress caused by the treatment with 131I could be reduced by using supplements such as vitamin C, vitamin E and selenium.
Elsendoorn et al. [125] (2001)	Yes	Europe	27 INT-g: 13 PL-g: 14	11.1%	Testicular, bladder, gastric, HNC and cervical cancers, sarcoma treated with cisplatin	ORALLY Selenium (100 mcg) vitamin E (400 mg) vitamin C (1000 mg)	7 days before the onset of CT until 3 weeks after cessation of therapy.	Before, during and after CT.	Acute and long-term genotoxicity, nephrotoxicity and ototoxicity induced by cisplatin.	Supplementation with vitamin C, vitamin E, and selenium did not reduce cisplatin-induced side effects.
Kim et al. [126] (2005)	Yes	America	48 INT-g: 39 PL-g: 9	100%	Prostate cancer	ORALLY Selenium (200 mcg/day) vitamin E (400 IU/die) vitamin C (250 mg/day)	3 to 6 weeks before prostatectomy.	NA	Serum proteomic patterns.	The mass profile showed that, after supplementation, proteomic patterns associated with a healthy status were found, before prostatectomy.
Hopkins et al. [127] (2010)	Yes	America	47	49%	Colorectal adenoma	ORALLY L-selenomethionine (200 mcg/day) Vitamin D (800 mg/day) vitamin C (1 g/day) β-carotene (24 mg/day) riboflavin (7.2 mg /day) niacin (80 mg/day) zinc (60 mg/day) manganese (5 mg/day)	Over 4 months.	Over 4 months	Oxidative and inflammatory biomarkers	In patients with colorectal adenoma, oxidative stress may decrease with the use of a cocktail of antioxidant micronutrients. However, this outcome must be related to the patient’s smoking status.
Weijl et al. [128] (2004)	Yes	Europe	48 INT-g: 23 PL-g: 25	14.6%	Testicular, gastrointestinal, urogenital, head and neck cancers, melanoma and osteosarcoma treated with cisplatin	ORALLY selenium (100 mcg/2 day) vitamin D (400 mg/day) vitamin C (1000 mg/day) protein (3.42 g/day) carbohydrate (7.92 g/day) fat (0.05 g/2 day)	7 days before until 3 weeks after cessation of chemotherapy.	2 and 12 months after ending of chemotherapy.	Nephrotoxicity and ototoxicity induced by cisplatin.	No protection against cisplatin-induced toxicities was found in enrolled cancer patients.
Federico et al. [129] (2001)	No	Europe	60 INT-g: 30 CTR-g: 30	38.3%	Colorectal carcinoma, gastric adenocarcinoma, esophageal carcinoma treated with CT	ORALLY Selenium (200 mg/day) Zinc (21 mg/day)	60 days	60 days	General clinical conditions.	Selenium and Zn supplementation can reduce asthenia and increase appetite in patients with gastrointestinal cancer.
Kazi et al. [130] (2012)	No	Asia	144	0%	Liver cancer	ORALLY Selenium (40 mcg/day) Zinc (30 mg/day)	60 days	60 days	Comparison of concentrations of As, Cd, Se, and Zn in serum and blood of liver cirrhosis.	The levels of Se and Zn are lower in liver cirrhosis/cancer patients than healthy subjects. In addition, the unhealthy group had As and Cf levels higher than age-matched reference subjects. A negative correlation between essential and toxic elements was observed.

Abbreviations: Natural Health Products, NHPs; Placebo group, PL-g; Intervention group, INT-g; Chemotherapy, CT; Cisplatin-induced peripheral neuropathy, CIPN; Total Neuropathy Score, TNS; Radiotherapy, RT; Paclitaxel-induced peripheral neuropathy, PIPN; Relative Risk, RR; Common Terminology Criteria for Adverse Events, CTCAE; Sensory Neuropathy, SN; Non-Small-Cell Lung Cancer, NSCLC; Peripheral Neuropathy, PNP; Oxaliplatin-induced peripheral neuropathy, OIPN; Acute Myeloid Leukemia, AML; Decitabine with cytarabine, aclarubicin hydrochloride, and granulocyte colony-stimulating factor, DCAG; Complete Remission, CR; Overall Survival, OS; Metastatic colorectal cancer, mCRC; Progression-free survival, PFS; Objective Response Rate, ORR; Cervical Intraepithelial Neoplasia, CIN; Colorectal cancer, CRC; Second primary cancers, SPC; Head and Neck cancer, HNC; Proliferating Cell Nuclear Antigen, PCNA; N-Acetylcysteine, NAC; Solid tumor, ST; Leukemia/lymphomas, LL; Planning Target Volume, PTV; Central Nervous System, CNS; Non–muscle-invasive bladder cancer, NMIBC; Natural Killer, NK; Quality of Life, QoL: Leucovorin calcium (folinic acid), fluorouracil, and oxaliplatin, FOLFOX.

## Data Availability

The data that support the findings of this study are openly available.
